# Risk of All-Cause Mortality in Alcohol-Dependent Individuals: A Systematic Literature Review and Meta-Analysis^☆^

**DOI:** 10.1016/j.ebiom.2015.08.040

**Published:** 2015-09-02

**Authors:** Philippe Laramée, Saoirse Leonard, Amy Buchanan-Hughes, Samantha Warnakula, Jean-Bernard Daeppen, Jürgen Rehm

**Affiliations:** a Université Claude Bernard Lyon I, 43 Boulevard du 11 Novembre 1918, 69100 Villeurbanne, France; b Lundbeck SAS, 37-45, Quai du Président Roosevelt, Issy-les-Moulineaux, 92445 Paris, France; c Costello Medical Consulting, City House, 126-130 Hills Road, Cambridge, CB2 1RE, UK; d Department of Public Health and Primary Care, University of Cambridge, 2 Worts' Causeway, Cambridge, CB1 8RN, UK; e University Alcohol Treatment Centre, Lausanne University Hospital, Rue du Bugnon 21, 1011 Lausanne, Switzerland; f Social and Epidemiological Research Department, Centre for Addiction and Mental Health, 33 Russell Street, Toronto, ON M5S 2S1, Canada; g Dalla Lana School of Public Health, University of Toronto, 155 College St, Toronto, ON M5T 3M7, Canada; h Klinische Psychologie und Psychotherapie, TU Dresden, Chemnitzer Str. 46, 01187 Dresden, Germany

**Keywords:** Alcohol dependence, Alcoholism, Abstinence, Mortality, Systematic review, Meta-analysis

## Abstract

**Background:**

Alcohol dependence (AD) carries a high mortality burden, which may be mitigated by reduced alcohol consumption. We conducted a systematic literature review and meta-analysis investigating the risk of all-cause mortality in alcohol-dependent subjects.

**Methods:**

MEDLINE, MEDLINE In-Process, Embase and PsycINFO were searched from database conception through 26th June 2014. Eligible studies reported all-cause mortality in both alcohol-dependent subjects and a comparator population of interest. Two individuals independently reviewed studies. Of 4540 records identified, 39 observational studies were included in meta-analyses.

**Findings:**

We identified a significant increase in mortality for alcohol-dependent subjects compared with the general population (27 studies; relative risk [RR] = 3.45; 95% CI [2.96, 4.02]; p < 0.0001). The mortality increase was also significant compared to subjects qualifying for a diagnosis of alcohol abuse or subjects without alcohol use disorders (AUDs). Alcohol-dependent subjects continuing to drink heavily had significantly greater mortality than alcohol-dependent subjects who reduced alcohol intake, even if abstainers were excluded (p < 0.05).

**Interpretation:**

AD was found to significantly increase an individual's risk of all-cause mortality. While abstinence in alcohol-dependent subjects led to greater mortality reduction than non-abstinence, this study suggests that alcohol-dependent subjects can significantly reduce their mortality risk by reducing alcohol consumption.

## Highlights

• A systematic review and meta-analysis were performed to investigate mortality risk in alcohol-dependent individuals.
• Alcohol dependence was associated with significantly higher mortality risk vs the general population, and vs alcohol abuse.
• Alcohol-dependent people with reduced alcohol consumption lowered their mortality risk, even if abstinence was not reached.

Individuals with alcohol dependence have a high risk of disease, disability or death. Treatment has traditionally focused on promoting abstinence, although some alcohol-dependent individuals would prefer to continue drinking in a controlled manner. By statistically combining results from previously published studies identified in a systematic literature review, we have shown that mortality among alcohol-dependent individuals is three to four times higher than in the general population. We have also found that individuals with alcohol dependence can reduce their risk of death by reducing alcohol consumption, even if they do not achieve abstinence.

## 1 Introduction

Alcohol use is one of the greatest risk factors for disease and disability (Rehm, 2011, Nutt et al., 2010, Rehm et al., 2009), and alcohol dependence (AD) seems to account for the majority of this burden (Rehm et al., 2012, Rehm et al., 2013). The risk of mortality has been shown to increase as alcohol consumption increases, both for lifetime risk and absolute annual risk, with absolute annual risk almost doubling as alcohol consumption increases from 10 g/day to 100 g/day (Rehm et al., 2011). In addition to the clinical burden of AD experienced by individuals (François et al., 2014), AD has wider societal consequences, including substantial direct and indirect economic costs (Rehm et al., 2012, Laramée et al., 2013).

Until the 1970s, alcohol use disorders (AUDs) were widely called ‘alcoholism’; by this time, however, it was apparent that AD could be considered as a separate diagnosis (Edwards and Gross, 1976). The current version of the *International Classification of Diseases* (ICD-10) continues to categorise harmful use and AD as separate diagnoses (World Health Organization, 1992), while the latest edition of the *Diagnostic and Statistical Manual of Mental Disorders* (DSM-5) has integrated alcohol abuse and AD into a single AUD diagnosis (American Psychiatric Association, 2013). In clinical practice, there is often no formal assessment of diagnoses (ie. alcohol abuse vs AD), but for treatment in specialised healthcare services it is safe to assume that most of the cases would qualify as the more severe form of AUD, corresponding to AD (Rehm et al., 2015a).

Previous systematic literature reviews (SLRs) and meta-analyses have examined the relative risk (RR) of all-cause or cause-specific mortality in people with AUDs compared with the general population or with controlled drinkers (Roerecke et al., 2013, Roerecke and Rehm, 2014, Roerecke and Rehm, 2013). One meta-analysis found an RR of 3.38 (95% CI [2.98, 3.84]) for men and 4.57 (95% CI [3.86, 5.42]) for women in clinical settings compared to the general population (Roerecke and Rehm, 2013); another found that individuals treated for AUDs reduced their mortality risk by more than half if they were able to reduce their alcohol consumption, compared to those individuals who continued to drink heavily (Roerecke et al., 2013). However, to our knowledge there are currently no systematic reviews focusing on the risk of all-cause mortality in alcohol-dependent individuals only.

Treatment for AD, and AUDs more widely, has traditionally focused on promoting abstinence as the only acceptable treatment goal. However, some patients may prefer a goal of non-problem drinking (Wallhed Finn et al., 2014). In recent years, there has been an increased emphasis on an alternative harm-reduction approach that attempts to help alcohol-dependent patients achieve a reduction in alcohol consumption without the need to completely abstain, consequently reducing the risk of harmful consequences associated with alcohol use (European Medicines Agency (EMA), 2010; National Institute for Health and Care Excellence (NICE), 2011). Reduced consumption of alcohol in individuals with AUDs has been shown to be beneficial, resulting in a significant reduction in mortality compared to continued heavy drinking (Roerecke et al., 2013), and is also predicted to improve the associated economic and societal burdens (Laramée et al., 2014).

In this study, we aimed to conduct an SLR and meta-analysis on the increased risk of all-cause mortality among individuals with AD compared to the general population, individuals without AUDs, and individuals qualifying for a diagnosis of alcohol abuse; and to examine the key factors affecting this risk. We also aimed to review the effect of reduced alcohol consumption among alcohol-dependent individuals.

## 2 Methods

### 2.1 Systematic Literature Review

An SLR was conducted in accordance with PRISMA guidelines (Moher et al., 2015) to identify studies reporting on mortality in alcohol-dependent subjects. MEDLINE, MEDLINE In-Process, Embase and PsycINFO were searched using the Ovid SP platform, and the Cochrane Library was searched using the Wiley Online platform. Search strings included terms relating to AD and mortality (Supplementary Tables 1 and 2). All searches were conducted on 26th June 2014; databases were searched for studies published from database conception up to that date.

Titles and abstracts of all studies identified in the database searches were screened using pre-defined eligibility criteria. Full texts for all potentially eligible studies were acquired and screened again. Screening at both stages was performed independently by two reviewers, with disagreements resolved by consensus or third-reviewer arbitration.

Studies were included if they were published in English and met the following criteria: they reported on subjects with AD; the study design was a randomised controlled trial (RCT), non-RCT, prospective observational study, retrospective cohort study, nested case–control study, systematic review or meta-analysis; mortality outcomes were reported for alcohol-dependent subjects; mortality in alcohol-dependent subjects was compared to mortality in an appropriate comparator population (including the general population, subjects without AUDs, subjects qualifying for a diagnosis of alcohol abuse, or alcohol-dependent subjects with differing levels of alcohol consumption); and a measure of association (hazard ratio [HR], odds ratio [OR], RR, standardised mortality ratio [SMR]) with 95% confidence intervals (CIs), or sufficient data to calculate these, was reported.

The “general population” comparator subgroup represented an unselected population of individuals in terms of drinking behaviour. This control group could therefore include a mixture of alcohol-dependent subjects, subjects qualifying for a diagnosis of alcohol abuse, abstinent subjects or individuals with any other level of pathological or non-pathological drinking. On the other hand, “subjects without AUDs” could be defined in a study as “non-alcoholics”, “subjects without AD or alcohol abuse diagnosis” or any similar definition.

Studies involving alcohol-dependent subjects were included irrespective of whether a formal definition of AD (e.g. ICD or DSM) had been used to identify them. For studies involving "alcoholics", the definition of alcoholism was reviewed to determine whether it was operationally similar to a diagnosis of AD (included) or alcohol abuse (excluded).

The reference lists of all included full texts were scanned for further potentially relevant studies. These studies then underwent full text review using the same criteria as studies identified in the database searches.

The study design, methodology, patient population parameters and outcomes for all studies included in the SLR were extracted into a pre-specified grid. Data extraction was performed by a single individual with independent verification by a second reviewer, with disagreements resolved by consensus or third-reviewer arbitration. It was planned that the quality and potential for bias of included RCTs would be assessed using the criteria provided by the York Centre for Reviews and Dissemination (Centre for Reviews and Dissemination, 2009) and the quality of non-RCTs would be assessed using the TREND checklist (Des Jarlais et al., 2004). The quality of observational studies was assessed using a checklist designed by the International Society for Pharmacoeconomics and Outcomes Research (ISPOR) Good Research Practices Taskforce, which includes domains for relevance and credibility. Credibility questions related to study design and data analysis, among others (Berger et al., 2014). Quality assessments were conducted from the perspective of the populations and outcomes of interest to this review. All studies found to be relevant and credible were eligible for meta-analysis.

### 2.2 Meta-Analyses

Meta-analyses were conducted in accordance with MOOSE guidelines (Stroup et al., 2000). Results from the included studies were pooled for meta-analysis by comparator population. Given the methodological heterogeneity of studies identified in this SLR (e.g. differences between studies in mean age, source of the alcohol-dependent population, and reference groups) a random-effects model was judged to be appropriate for this meta-analysis (parallel analyses used fixed-effect models). HRs, ORs, RRs and SMRs were assumed to approximate the same measure of risk (Rothman and Greenland, 1998).

Included studies were pooled for meta-analyses based on measures of association being available for the following comparisons: alcohol-dependent subjects vs the general population, subjects without AUDs, or alcohol abusing subjects; or alcohol-dependent subjects who continued to drink heavily vs alcohol-dependent subjects who reduced their alcohol intake (abstainers excluded), alcohol-dependent subjects who reduced their alcohol intake (abstainers included), or abstinent alcohol-dependent subjects.

Where the same patients were included in two or more studies, the study involving the greatest number of alcohol-dependent subjects was included in the meta-analysis.

To test the robustness of the findings, subgroup meta-analyses were performed within studies that compared alcohol-dependent subjects vs the general population, by a number of pre-specified study- and patient-level characteristics. This included a subgroup analysis by the definition of AD used within the study (strictly defined AD, such as DSM or ICD criteria vs another definition vs no reported definition). It was not possible to perform subgroup analyses for the other comparison groups due to the limited number of studies and insufficient power.

Random-effects were estimated using the approach of DerSimonian and Laird (1986), with the estimate of heterogeneity being taken from the Mantel–Haenszel model (Mantel and Haenszel, 1959). Summary RRs and 95% CIs were calculated by pooling the study-specific estimates. Stata® statistical software was used, in particular, the METAN command written for Stata® v11.1 (Anon, 2013). Consistency of findings across individual studies was assessed by standard χ^2^ tests and the I^2^ statistic (Higgins et al., 2003). Statistical tests were two-sided and used a significance level of p < 0.05. Publication bias was assessed by funnel plots and Egger's tests (Egger et al., 1997).

### 2.3 Role of the Funding Source

The study sponsor was involved in the design of the review and its protocol, the review of the data collection and statistical analysis, and the interpretation of the results. The study sponsor was not involved in the collection or extraction of data, or in the performance of statistical analyses. All authors were involved in the writing of the report and the decision to submit the manuscript for publication.

## 3 Results

**Fig. 1:**
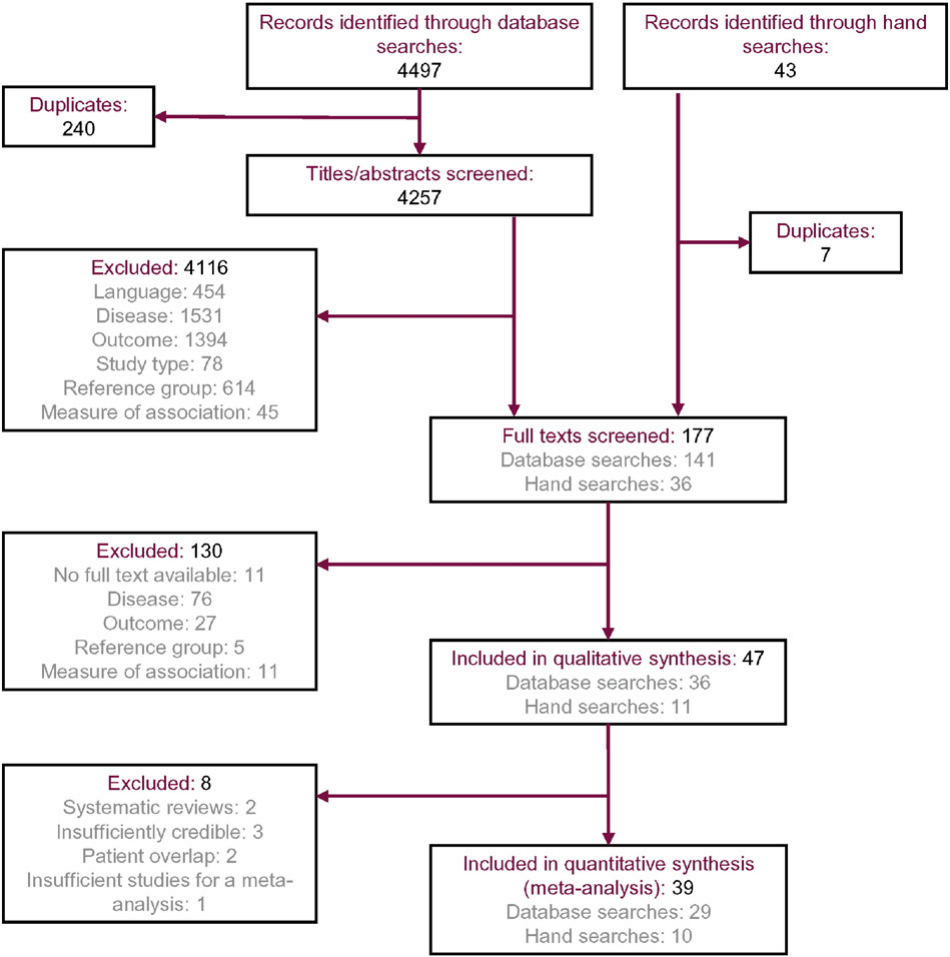
PRISMA flowchart.

Of the 4540 records identified through the database searches and hand searches, 177 were selected for full-text review and 47 (including two systematic reviews (Roerecke and Rehm, 2014, Roerecke and Rehm, 2013) and 45 primary research studies) were ultimately included in the qualitative synthesis of results (Fig. 1). Of the 45 primary research studies, three were unsuitable for inclusion in the quantitative synthesis. Of these, two studies duplicated data on subjects from other included studies (de Lint and Schmidt, 1970, Martin et al., 1985a). The third study was excluded since it compared alcohol-dependent subjects vs abstinent subjects without AUDs; no other studies included in the SLR reported an equivalent comparison, so a meta-analysis was not conducted (Dawson, 2000).

Following a thorough quality appraisal, results from three studies were judged to be insufficiently credible for meta-analysis due to a lack of information presented about key components of their protocols (Bell and Orjasaeter, 1983, Fitzgerald et al., 1971, Kessel and Grossman, 1961). The quality of the included studies varied substantially. Frequently-encountered limitations included the lack of a reported definition for AD and results presented without confidence intervals or without adjustment for confounders. Summaries of the credibility domain of the quality appraisals are presented in Supplementary Table 3 for all studies included in the SLR. Thirty-nine studies included in the SLR were ultimately eligible for meta-analysis (Table 1).

The majority of the studies included in the meta-analyses (28/39) involved patients selected from AD treatment facilities (Berglund and Tunving, 1985, Campos et al., 2011, de Lint and Levinson, 1975, Denison et al., 1997, Feuerlein et al., 1994, Finney and Moos, 1991, Gerdner and Berglund, 1997, Gillis, 1969, Gual et al., 2009, Haver et al., 2009, Hiroeh et al., 2008, Johnson, 2001, Mackenzie et al., 1986, Marshall et al., 1994, Martin et al., 1985b, Moos et al., 1994, Noda et al., 2001, Rankin et al., 1970, Saieva et al., 2012, Schmidt and de Lint, 1969, Smith et al., 1983, Storbjörk and Ullman, 2012, Tashiro and Lipscomb, 1963, Thorarinsson, 1979, Vaillant et al., 1983, Wells and Walker, 1990, Yoshino et al., 1997, De Silva and Ellawala, 1994). Two studies involved patients from hospital populations (Poser et al., 1992, Wallerstedt et al., 1995), although they were not necessarily being treated for AD. Eight studies identified alcohol-dependent subjects from general population surveys (Mattisson et al., 2011, John et al., 2013, Markkula et al., 2012, Min et al., 2008, Murphy et al., 2008, Neumark et al., 2000, Perälä et al., 2010, Vaillant, 2003), and a single study was based on a survey of company employees (Pell and D'Alonzo, 1973). Only four studies included in the meta-analyses reported mean daily or weekly alcohol consumption among alcohol-dependent subjects (Finney and Moos, 1991, Haver et al., 2009, Johnson, 2001, Perälä et al., 2010).

**Fig. 2:**
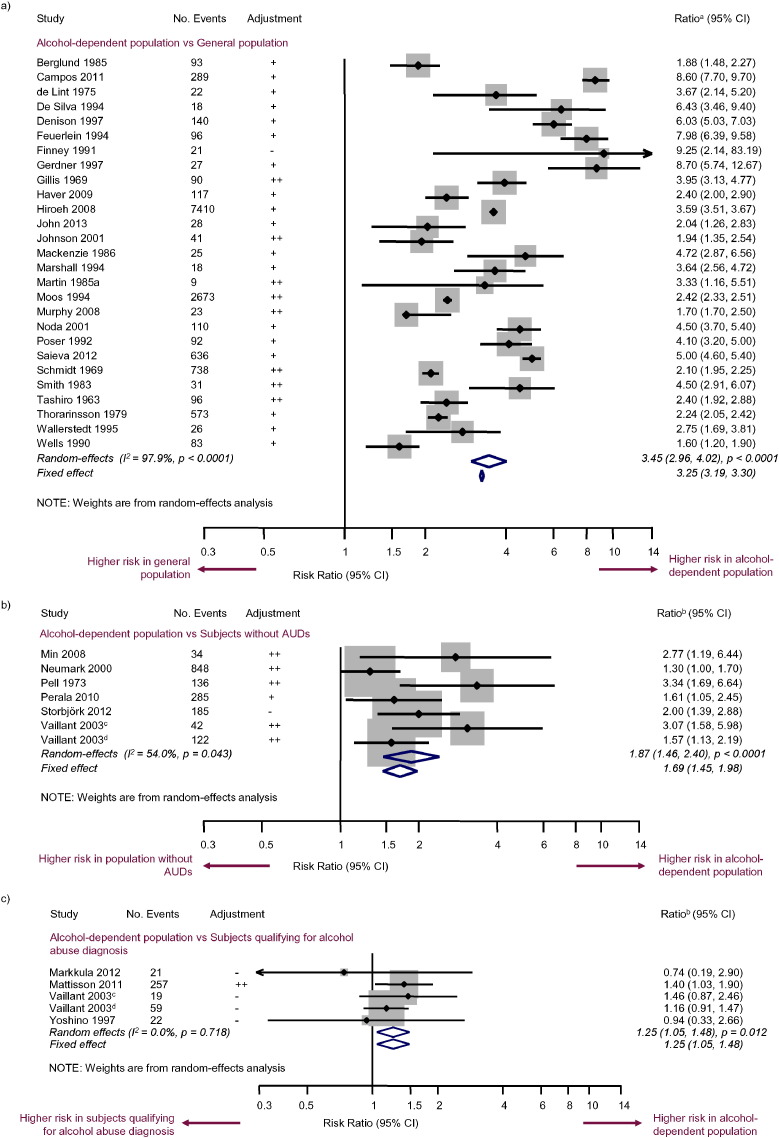
Random-effects and fixed-effect meta-analyses of mortality risk in a) alcohol-dependent subjects vs the general population, b) alcohol-dependent subjects vs subjects without AUDs, and c) alcohol-dependent subjects vs subjects qualifying for a diagnosis of alcohol abuse. ^a^HR, OR, RR or SMR (depending on study); ^b^HR, OR or RR (depending on study); ^c^College cohort; ^d^Core city cohort.

The most commonly reported comparison was alcohol-dependent subjects vs the general population, which was reported in 27/39 of the studies eligible for meta-analysis, with a total of 13,523 deaths in the alcohol-dependent populations (Fig. 2a). The majority of studies in this category determined the expected death rate of the alcohol-dependent sample based on age- and sex-matched data from demographic records from the city or country of the study. The pooled RR calculated from these studies was 3.45 (95% CI [2.96, 4.02]; p < 0.0001). Heterogeneity between studies was high (I^2^ = 97.9%; p < 0.0001).

**Fig. 3:**
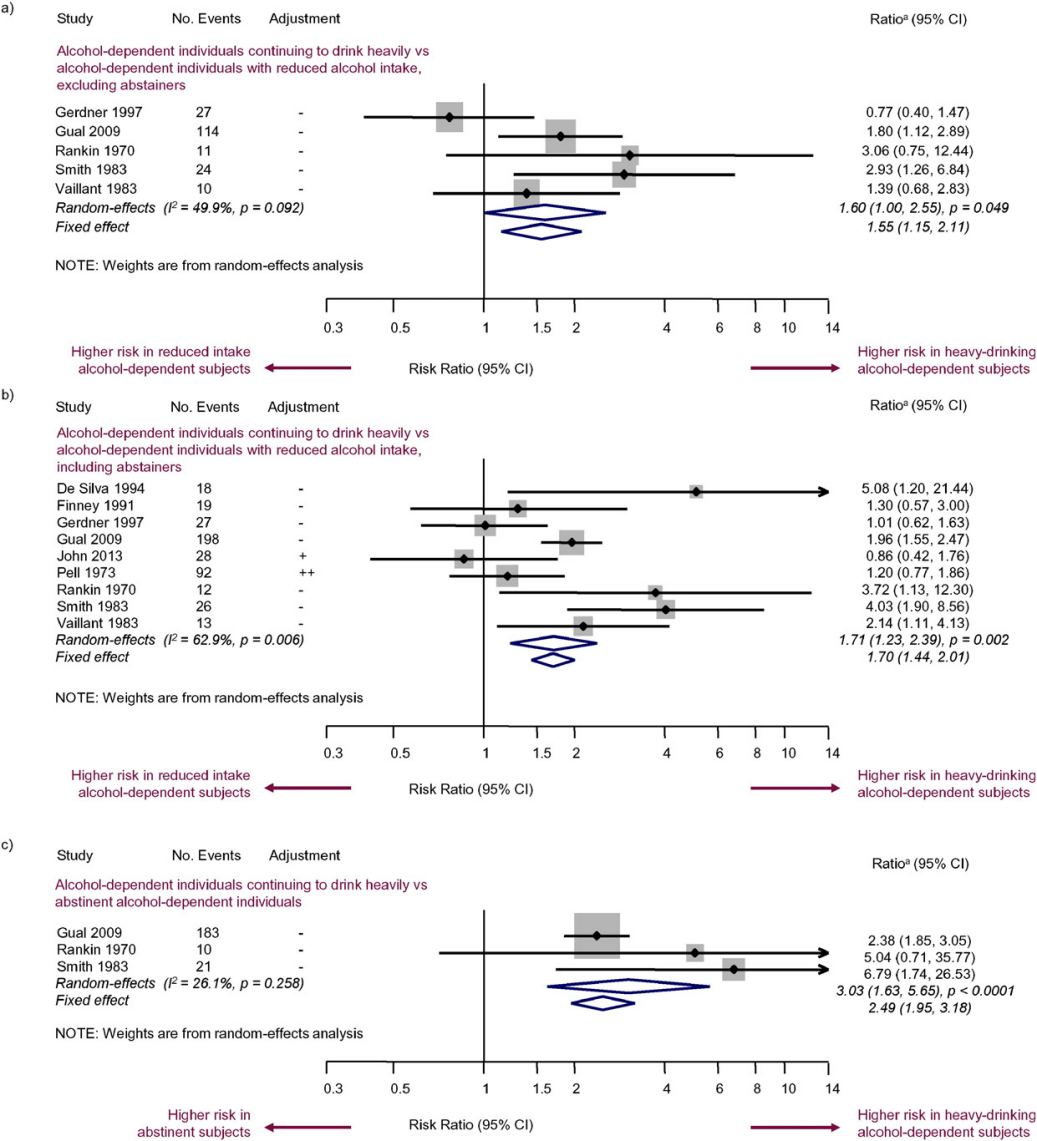
Random-effects and fixed-effect meta-analyses of mortality risk in alcohol-dependent subjects with continued heavy drinking vs a) alcohol-dependent subjects who reduced their alcohol intake (excluding abstainers), b) alcohol-dependent subjects who reduced their alcohol intake (including abstainers), and c) abstinent alcohol-dependent subjects. ^a^HR, OR or RR (depending on study).

Other all-cause mortality comparisons investigated in the meta-analyses included: alcohol-dependent subjects vs subjects without AUDs (six studies; RR = 1.87; 95% CI [1.46, 2.40]; p < 0.0001) (Fig. 2b); alcohol-dependent subjects vs subjects qualifying for a diagnosis of alcohol abuse (four studies; RR = 1.25; 95% CI [1.05, 1.48]; p = 0.012) (Fig. 2c); alcohol-dependent subjects who continued to drink heavily vs alcohol-dependent subjects who reduced their alcohol intake, excluding abstainers (five studies; RR = 1.60; 95% CI [1.00, 2.55]; p = 0.049) (Fig. 3a); alcohol-dependent subjects who continued to drink heavily vs alcohol-dependent subjects who reduced their alcohol intake, including abstainers (nine studies; RR = 1.71; 95% CI [1.23, 2.39]; p = 0.002) (Fig. 3b); and alcohol-dependent subjects who continued to drink heavily vs abstinent alcohol-dependent subjects (three studies; RR = 3.03; 95% CI [1.63, 5.65]; p < 0.0001) (Fig. 3c).

Subgroup analyses within studies that compared alcohol-dependent subjects with the general population (Supplementary Fig. 1) indicated evidence of heterogeneity by level of adjustment and length of follow-up (p-value from meta-regression on each covariate; p = 0.02 and p = 0.03, respectively); a lower level of adjustment or a shorter follow-up corresponded to a higher RR. Furthermore, there was a noticeable difference in mortality between alcohol-dependent subjects selected from general population surveys (RR = 1.76; 95% CI [1.48, 2.09]) and alcohol-dependent subjects selected from treatment centres (RR = 3.65; 95% CI [3.10, 4.30]) or hospitals (RR = 3.49; 95% CI [2.37, 5.12]). The definition of AD used in a study (DSM or ICD AD vs other definition vs no reported definition) did not have a significant effect on the RR (p = 0.86).

There was no evidence of publication bias across studies that reported on alcohol-dependent subjects vs the general population, alcohol-dependent subjects vs subjects qualifying for a diagnosis of alcohol abuse, or alcohol-dependent subjects with continued heavy drinking vs alcohol-dependent subjects who reduced their drinking (p > 0.05 in Egger's asymmetry test). However, there was evidence of publication bias for analyses that compared alcohol-dependent subjects to subjects without AUDs (Egger's asymmetry test of associations: p = 0.01) (Supplementary Fig. 2).

## 4 Discussion

In these meta-analyses, alcohol-dependent individuals were found to have a significantly increased risk of all-cause mortality compared to the general population. However, heterogeneity between studies was high. While in subgroup analyses the definition of AD reported in a study (strict AD vs any other definition vs no definition reported) did not affect the pooled RR estimates, differing levels of adjustment between studies (e.g. adjustment for age, sex and smoking vs unadjusted comparisons) and length of follow-up were identified as significant contributors to heterogeneity.

The general population includes individuals with AUDs, and would therefore be expected to have higher alcohol-attributable mortality than a cohort without AUDs. The relative risk of mortality for alcohol-dependent subjects vs the general population should therefore be lower than the relative risk for alcohol-dependent subjects vs a cohort without AUDs. However, in this study the opposite was found; a higher risk estimate for studies comparing alcohol-dependent subjects to the general population than for studies comparing alcohol-dependent subjects to cohorts without AUDs. This may be explained by considering the characteristics of the alcohol-dependent population in each case: of the studies reporting on mortality in alcohol-dependent subjects vs the general population, the majority involved treated alcohol-dependent subjects. However, of the six studies reporting on mortality in alcohol-dependent subjects vs subjects without AUDs, only one study involved treated alcohol-dependent subjects. Subgroup analyses of studies comparing alcohol-dependent subjects to the general population showed that the pooled risk estimate for studies involving treated alcohol-dependent subjects was higher than for studies involving alcohol-dependent subjects identified from general population surveys, potentially explaining the result above. This reasoning can be further strengthened by recent studies on characteristics of treated vs untreated people with AUDs: there was a substantial difference with respect to co-morbid conditions (both for mental and somatic disorders), alcohol consumption (both average drinking levels and binge drinking episodes), hospital days and disability (Rehm et al., 2015a, Rehm et al., 2014).

Although only one out of four studies reporting on mortality in alcohol-dependent individuals compared to subjects qualifying for a diagnosis of alcohol abuse showed significantly increased mortality for alcohol-dependent subjects (Mattisson et al., 2011), the pooled RR was significant. Reduced alcohol consumption without abstinence was significantly associated with a lower risk of death in alcohol-dependent subjects. Although this was a lower reduction in mortality than for abstinent alcohol-dependent subjects, the opportunity to reduce the risk of death combined with the preference among some patients to achieve a non-problem drinking outcome rather than abstinence supports the use of controlled drinking as a valid AD treatment goal (Adamson et al., 2010, Luquiens et al., 2011, Rosenberg et al., 1992). Achieving a reduction in alcohol consumption, including among alcohol-dependent subjects who may otherwise not have entered into treatment, would be predicted to ease the overall public health burden associated with AD (François et al., 2014), as well as reducing both direct and indirect costs (Rehm et al., 2012, Laramée et al., 2014).

The results of these meta-analyses are consistent with recently published meta-analyses on the risk of mortality in individuals with AUDs. Roerecke and Rehm (2013) reported RRs of 3.38 (95% CI [2.98, 3.84]) and 1.91 (95% CI [1.51, 2.42]) for men with AUDs identified from clinical samples and population surveys, respectively, compared to the general population (Roerecke and Rehm, 2013). This is broadly in agreement with the findings of our subgroup analyses, where the pooled RR for treated alcohol-dependent subjects was more than double the pooled RR for alcohol-dependent subjects from population surveys. Although this may seem counterintuitive, only a minority of individuals with substance dependence seek treatment for their addiction, and these individuals tend to have a greater severity of dependence than those who do not seek help (Grella and Stein, 2013). This may explain the increased mortality among treated alcohol-dependent subjects. Our observations that studies with shorter follow-up periods or with results adjusted for fewer confounders reported higher RRs were also consistent with previously published results (Roerecke and Rehm, 2013).

In clinical practice, the distinction between alcohol abuse, AD and AUDs is not always clear. However, given the low treatment rates, and the comparisons of treated vs untreated individuals with AUDs (Rehm et al., 2015a, Rehm et al., 2014), it is reasonable to assume that the majority of people in specialised treatment qualify for a diagnosis of AD (Rehm et al., 2015b). The definition of AD was carefully considered in the eligibility criteria for this SLR, and the impact of including studies where formal definitions (such as ICD or DSM) had not been used to identify alcohol-dependent subjects was subsequently evaluated in sub-group analyses and found to not have a significant effect. These factors may explain why the results of this SLR on AD are similar to previously published SLRs on AUDs (Roerecke and Rehm, 2013, Harris and Barraclough, 1998). To our knowledge, there are no previous SLRs or meta-analyses reporting on mortality in alcohol-dependent individuals specifically rather than individuals with AUDs more broadly. Our study therefore supplements the existing literature on mortality in individuals with AUDs (Roerecke et al., 2013, Roerecke and Rehm, 2014, Roerecke and Rehm, 2013), supporting the conclusions of previous studies while providing an important focus on the subgroup of subjects with AD.

One limitation of this study was the exclusion of non-English language articles; however, in our subgroup analysis of the studies comparing alcohol-dependent subjects vs the general population – half of which were from Europe, a third from North America and the remainder from other locations – there were no significant differences between regions, implying that the inclusion of further studies from other countries or continents would not necessarily have significantly changed the conclusions of this review. Another limitation is that we did not exclude unadjusted studies from the meta-analyses. This may have affected some results, as unadjusted studies were found to show a significantly larger effect size in the comparison between alcohol-dependent subjects and the general population. However, we were unable to conduct subgroup analyses by adjustment level for the other meta-analyses due to the limited number of studies and insufficient power. Therefore, it is not possible to determine the direction of any potential effects in these cases. Finally, it is possible that the risk of mortality in alcohol-dependent subjects may vary by how long an individual has been alcohol-dependent for; however, we were unable to investigate this, as this information was not commonly reported in the included studies.

In our meta-analyses, we have established that alcohol-dependent individuals who are able to reduce their alcohol consumption reduce their risk of all-cause mortality, but without further insight into the causes of death among these individuals we can only speculate about the mechanism by which this reduction in mortality occurs. It is not clear whether a reduction in alcohol consumption among these individuals would lead to a proportional reduction in risk across all causes of death, or whether the benefits would be greater among specific causes. Furthermore, though a meta-analysis has been published on cause-specific mortality in individuals with AUDs (Roerecke and Rehm, 2014), none has been published concerning cause-specific mortality in individuals with AD specifically. We therefore recommend that further research should focus on the risk of cause-specific mortality in alcohol-dependent individuals, and particularly in individuals who are able to reduce their alcohol consumption.

## 5 Conclusions

We present the finding that AD significantly increases an individual's risk of all-cause mortality compared to the general population, subjects without AUDs or subjects qualifying for a diagnosis of alcohol abuse. While abstinence in alcohol-dependent subjects leads to a greater reduction in mortality than non-abstinence, alcohol-dependent subjects who are able to reduce their alcohol consumption can significantly reduce their risk of death. This finding reinforces the validity of harm reduction and the associated societal benefit through reduced alcohol consumption as a valid treatment goal alongside abstinence in the treatment of AD.

## Author Contributions

Study design and protocol development: All authors.

Collection and extraction of data: Leonard, Buchanan-Hughes.

Statistical analysis: Warnakula.

Review of the data collection and of the statistical analysis, technical support and supervision: Laramée, Leonard, Rehm.

Administrative or material support: Laramée, Leonard, Buchanan-Hughes.

Interpretation of data and drafting of the manuscript: All authors.

Critical revision of the manuscript for important intellectual content: All authors.

Obtained funding: Laramée.

## Conflict of Interest Disclosures

All authors have completed and submitted the ICMJE Form for Disclosure of Potential Conflicts of Interest. Dr. P. Laramée was an employee of Lundbeck SAS at the time this study was conducted; Ms. S. Leonard and Ms. A. Buchanan-Hughes are employees of Costello Medical Consulting, which was contracted by Lundbeck SAS to support this study; Dr. S. Warnakula received personal fees from Costello Medical Consulting during the conduct of this study; Prof. J.-B. Daeppen received personal fees from Lundbeck SAS during the conduct of this study; Prof. J. Rehm has received grants, personal fees and non-financial support from Lundbeck SAS, outside the submitted work.

## Funding and Support

This study was funded by Lundbeck SAS.

## Additional Contributions

The authors acknowledge Artur Arikainen (Costello Medical Consulting, UK) for writing and editorial assistance.

## Figures and Tables

**Table 1:** Characteristics of the 39 primary research studies on AD and all-cause mortality included in the meta-analyses.

Study	Sex	Location	Study design	Baseline age of subjects (years)	Time period	Maximum follow-up (years)	Number of alcohol-dependent individuals	Setting and subjects	Definition of AD	Comparison groups used
Berglund and Tunving (1985)	M	Sweden	Prospective cohort	Mean 42	1960 to 1980	20	257	Alcoholics treated at the outpatient alcoholic unit at the University Hospital, Lund	NR	AD vs general population
Campos et al. (2011)	M, F	Spain	Retrospective cohort	18 to 89	1996 to 2006	10	1265	Admissions to a university hospital in Spain who were either admitted for alcohol withdrawal syndrome (AWS) or developed AWS during admission	ICD-9-CM AWS	AD vs general population
de Lint and Levinson (1975)	M, F	Canada	Prospective cohort	20 to 74	1969 to 1974	5	154	Admissions to the Donwood Institute who were addicted primarily to alcohol	Addicted primarily to alcohol	AD vs general population
De Silva and Ellawala (1994)	M	Sri Lanka	Prospective cohort	Mean 39	1986 to 1991	6	188	Alcohol-dependent men admitted to the Sumithrayo Rehabilitation Unit	World Health Organization (WHO) 1951 definition (“an excessive intake of alcohol leading to physical, psychological or social harm”)	AD vs general population; AD with continued heavy drinking vs reduced intake
Denison et al. (1997)	M	Sweden	Prospective cohort	≥ 20	1986 to 1991	5	1049	Alcohol-dependent men treated as inpatients in the detoxification ward at the University Psychiatric Clinic, Lillhagen Hospital, Göteborg	DSM-III-R AD	AD vs general population
Feuerlein et al. (1994)	M, F	Germany	Prospective cohort	≥ 20	1981 to 1985	4	1401	Alcoholics treated at 21 different treatment centres in West Germany	NR	AD vs general population
Finney and Moos (1991)	M, F	USA	Prospective cohort	NR	NR	8	113	Alcoholic patients treated in one of five residential facilities who returned to family settings	NR	AD vs general population; AD with continued heavy drinking vs reduced intake
Gerdner and Berglund (1997)	M, F	Sweden	Prospective cohort	26 to 63	1985 to 1994	8.5	116	Patients competing a five-week Alcoholics Anonymous-oriented programme at Runnagarden Social Welfare Institution	AD (diagnostic criteria NR)	AD vs general population; AD with continued heavy drinking vs reduced intake
Gillis (1969)	M, F	South Africa	Prospective cohort	20 to 74	1959 to 1965	6	802	Chronic alcoholic white patients admitted to three treatment centres in South Africa	Chronic addictive alcoholics	AD vs general population
Gual et al. (2009)	M, F	Spain	Prospective cohort	18 to 55	1987 to 2008	20	850	Alcohol-dependent patients who had accepted to enter a treatment programme, and who had a stable home with at least one other family member	DSM-III AD	AD with continued heavy drinking vs reduced intake
Haver et al. (2009)	F	Sweden	Prospective cohort	Mean 42.5	1981 to 2007	25	420	Subjects receiving their first treatment at the Early Treatment for Women with Alcohol Addiction programme at Karolinska Hospital, Stockholm	Alcohol addiction	AD vs general population
Hiroeh et al. (2008)	M, F	Denmark	Retrospective cohort	≥ 15	1973 to 1993	21	NR (275,874 person-years)	All Danish adults aged 15 years or over, identified through the Danish Civil Registration System	ICD-8 alcoholism	AD vs general population
John et al. (2013)	M, F	Germany	Prospective cohort	18 to 64	1996 to 2010	14	147	Random sample of the general population of Germany	DSM-IV AD	AD vs general population; AD with continued heavy drinking vs reduced intake
Johnson (2001)	M, F	UK	Retrospective cohort	47 to 74	1978 to 1998	20	100	Subjects who attended the Robert Smith Unit, a day centre for the treatment of alcohol problems in Bristol, as part of their first referral for treatment	ICD-10 AD	AD vs general population
Mackenzie et al. (1986)	M	USA	Prospective cohort	Mean 41	1969 to 1979	8.2	85	Male alcoholics who had participated in the Francis Scott Key Medical Center inpatient alcoholism research programme	NR	AD vs general population
Markkula et al. (2012)	M, F	Finland	Prospective cohort	30 to 70	2000 to 2008	8	6372	Participants in the Health 2000 Study, a nationally representative sample of Finnish people	DSM-IV AD	AD vs alcohol abuse
Marshall et al. (1994)	M	UK	Prospective cohort	39 to 43	1968 to 1990	20	99	Married men with a diagnosis of alcoholism but no psychotic illness, who attended the specialist alcohol problems clinic at Maudsley Hospital, London	NR	AD vs general population
Martin et al. (1985b)	M, F	USA	Prospective cohort	14 to 84	1967 to 1979	12	70	Outpatients admitted to the Washington University Psychiatry Clinic	Similar to Feighner Criteria	AD vs general population
Mattisson et al. (2011)	M, F	Sweden	Prospective cohort	Median 27 (M)/16 (F)	1947 to 1997	50	208	The Lundby Cohort, comprising all subjects living in Lundby District	DSM-IV AD	AD vs alcohol abuse
Min et al. (2008)	M, F	South Korea	Nested case–control	22 to 82	1998 to 2004	6	59	Adults living in Guyrae-myon village	≥ 16 on the Severity of Alcohol Dependence Questionnaire (SADQ) for men; ≥ 10 on the SADQ for women	AD vs no AUDs
Moos et al. (1994)	M, F	USA	Prospective cohort	≥ 55	1986 to 1991	4	12,309	AD patients in Department of Veterans Affairs (VA) Medical Centers	ICD-9-CM AD	AD vs general population
Murphy et al. (2008)	M, F	Canada	Prospective cohort	18 to 88	1952 to 1992	40	NR	Heads of household in Stirling County	Psychiatric diagnosis of alcoholism with high confidence	AD vs general population
Neumark et al. (2000)	M, F	USA	Prospective cohort	≥ 18	1981 to 1996	14	284	Adult household residents living in the Baltimore Epidemiologic Catchment Area	DSM-III AD	AD vs no AUDs
Noda et al. (2001)	M	Japan	Prospective cohort	21 to 77	1972 to 1992	20	306	Patients diagnosed with alcoholism at a psychiatric institution	Alcoholism (Japanese Committee for the Diagnosis of Alcoholism criteria)	AD vs general population
Pell and D'Alonzo (1973)	M, F	USA	Prospective cohort	Median 51.1	1963 to 1969	5	899	Active or retired (with pension) employees of the DuPont company	Persons who exhibit alcohol dependency, drinking patterns and behavioural characteristics such as disturbed personal relations and impaired work efficiency, that clearly demonstrate they are chronic, uncontrolled alcoholics	AD vs no AUDs; AD with continued heavy drinking vs reduced intake
Perälä et al. (2010)	M, F	Finland	Prospective cohort	≥ 30	2000 to 2008	8	443	Participants in the Health 2000 Study, a nationally representative sample of Finnish people	DSM-IV AD	AD vs no AUDs
Poser et al. (1992)	M, F	Germany	Prospective cohort	Mean 28.7	1974 to 1991	17	620	Patients with known AD who had any contact with the psychiatric or neurological department of the University Hospital of Göttingen for therapy or expert opinion	DSM-III AD	AD vs general population
Rankin et al. (1970)	M, F	Australia	Prospective cohort	NR	1964 to 1969	4.75	56	Alcoholics with cirrhosis, attending the Alcoholism Clinic at St. Vincent's Hospital, Melbourne	NR	AD with continued heavy drinking vs reduced intake
Saieva et al. (2012)	M, F	Italy	Prospective cohort	14 to 93	1985 to 2006	21.7	2272	Alcoholic residents of Tuscany, treated at the Alcohol Centre of Florence	ICD-9 AD	AD vs general population
Schmidt and de Lint (1969)	M, F	Canada	Prospective cohort	≥ 15	1951 to 1966	14	6514	Patients admitted to the Toronto Clinic of the Addiction Research Foundation	NR	AD vs general population
Smith et al. (1983)	F	USA	Prospective cohort	18 to 67	1967 to 1980	11	103	Women diagnosed with alcoholism at two psychiatric hospitals in the St. Louis area	Feighner Criteria	AD vs general population; AD with continued heavy drinking vs reduced intake
Storbjörk and Ullman (2012)	M, F	Sweden	Prospective cohort	Mean 43.3	2000 to 2008	8	929	Patients from 21 treatment units, who started treatment for AD that they had not previously been given at the same treatment unit during the previous six months	ICD-10 AD	AD vs no AUDs
Tashiro and Lipscomb (1963)	M, F	USA	Prospective cohort	20 to 79	1954 to 1958	5	1692	Individuals admitted to four alcoholism treatment facilities in California	NR	AD vs general population
Thorarinsson (1979)	M	Iceland	Prospective cohort	Mean 37	1951 to 1974	23	2863	First-admission alcoholic males treated as either an outpatient or an inpatient at one of three clinics, or identified as attending a private clinic by the Psychiatric Register of Iceland	NR	AD vs general population
Vaillant et al. (1983)	M, F	USA	Prospective cohort	NR	1972 to 1980	8	110	Patients admitted for alcohol withdrawal to the inpatient ward at the Cambridge and Somerville Program for Alcohol Rehabilitation at the Cambridge Hospital	Patients with alcohol withdrawal who required ≥ 750 mg of chlorodiazepoxide during detoxification or who revealed signs of severe withdrawal such as seizures or delirium tremens during prior admissions	AD with continued heavy drinking vs reduced intake
Vaillant (2003)	M	USA	Prospective cohort	9 to 20	1940 to 2000	23	91	College cohort: Male Harvard University sophomores selected for a study of normal development, with no known physical or mental illness at baseline Core city cohort: Men studied from early adolescence as a non-delinquent community control group for a study of institutionalised juvenile delinquents	DSM-III AD	AD vs no AUDs; AD vs alcohol abuse
Wallerstedt et al. (1995)	M	Sweden	Prospective cohort	17 to 79	1980 to 1987	7	52	Patients in the medical, surgical and orthopaedic wards of the Ostra Hospital, Göteborg	Patients with presence of one or more of the following criteria: interview reports of altered reactions to alcohol or notes on alcohol addiction in their case files; treatment for alcohol addiction at a psychiatric clinic; registration by the social authorities for alcohol addiction	AD vs general population
Wells and Walker (1990)	M, F	New Zealand	Prospective cohort	≥ 15	1972 to 1984	11	616	Alcoholic patients admitted to Mahu Clinic, Sunnyside Hospital, Christchurch	NR	AD vs general population
Yoshino et al. (1997)	M	Japan	Prospective cohort	Mean 50.1	1989 to 1996	3	74	Alcoholics hospitalised at the Komagino Hospital Alcoholism Treatment Unit for a detoxification and rehabilitation programme	DSM-III AD	AD vs alcohol abuse

AD, alcohol dependence; AWS, alcohol withdrawal syndrome; DSM, Diagnostic and Statistical Manual of Mental Disorders; F, female; ICD, International Classification of Diseases; M, male; NR, not reported; SADQ, Severity of Alcohol Dependence Questionnaire; WHO, World Health Organization.
