# Supplementary material for: Risk of All-Cause Mortality in Alcohol-Dependent Individuals: A Systematic Literature Review and Meta-Analysis
Source: eBioMedicine. 2015 Sep 2;2(10):1394–404. doi: 10.1016/j.ebiom.2015.08.040 (PMC4634361; doi:10.1016/j.ebiom.2015.08.040)

Supplementary Table 1. Search strategy for MEDLINE, MEDLINE In-Process, Embase and PsycINFO (searched simultaneously via the Ovid SP platform)

| **Term group** | **Search number** | **Search terms** |
| --- | --- | --- |
| **Exposure** | 1 | exp Alcoholism/ |
|  | 2 | “Alcohol dependen$”.tw. |
|  | 3 | “Alcohol use disorder$”.tw. |
|  | 4 | “Alcohol misuse”.tw. |
|  | 5 | Alcoholic$.tw. |
|  | 6 | Alcoholism.tw. |
|  | **7** | **Or/1-6** |
| **Outcome** | 8 | exp Death/ |
|  | 9 | Death$.tw. |
|  | 10 | exp Mortality/ |
|  | 11 | Mortalit$.tw. |
|  | **12** | **Or/8-11** |
| **Study Design** | 13 | exp Randomized Controlled Trials as Topic/ |
|  | 14 | exp Randomized Controlled Trial/ |
|  | 15 | exp Random Allocation/ |
|  | 16 | exp Randomization/ |
|  | 17 | exp Double Blind Method/ |
|  | 18 | exp Single Blind Method/ |
|  | 19 | exp Single Blind Procedure/ |
|  | 20 | exp Double Blind Procedure/ |
|  | 21 | exp Crossover Procedure/ |
|  | 22 | ((singl$ or doubl$ or treb$ or tripl$) adj (blind$3 or mask$3)).tw. |
|  | 23 | exp Clinical Trial/ |
|  | 24 | Clinical Trials as Topic/ |
|  | 25 | Clinical Trial, Phase II.pt. |
|  | 26 | Clinical Trial, Phase III.pt. |
|  | 27 | Clinical Trial, Phase IV.pt. |
|  | 28 | exp Phase 2 Clinical Trial/ |
|  | 29 | exp Phase 3 Clinical Trial/ |
|  | 30 | exp Phase 4 Clinical Trial/ |
|  | 31 | Controlled clinical trial.pt. |
|  | 32 | Randomized controlled trial.pt. |
|  | 33 | Multicenter study.pt. |
|  | 34 | Clinical trial.pt. |
|  | 35 | (Clinical adj (trial$ OR stud$)).tw. |
|  | 36 | exp Placebos/ |
|  | 37 | exp Placebo/ |
|  | 38 | Placebo$.ti,ab. |
|  | 39 | “Randomly allocated”.tw. |
|  | 40 | (Allocated adj2 random$).tw. |
|  | 41 | Random allocation.tw. |
|  | 42 | RCT.tw. |
|  | 43 | ((“Randomi?ed controlled”) adj (trial$ or stud$)).tw. |
|  | 44 | Open-label trial$.tw. |
|  | 45 | Open-label stud$.tw. |
|  | 46 | Non-blinded stud$.tw. |
|  | 47 | exp Cohort Studies/ |
|  | 48 | exp Cohort Analysis/ |
|  | 49 | Cohort analy$.tw. |
|  | 50 | (Cohort adj (study or studies)).tw. |
|  | 51 | exp Longitudinal Studies/ |
|  | 52 | exp Longitudinal Study/ |
|  | 53 | Longitudinal.tw. |
|  | 54 | exp Follow-Up Studies/ |
|  | 55 | exp Follow-Up/ |
|  | 56 | (Follow up adj (study or studies)).tw. |
|  | 57 | exp Prospective Studies/ |
|  | 58 | exp Prospective Study/ |
|  | 59 | (Prospective adj (cohort$ or stud$)).tw. |
|  | 60 | exp Nested Case Control Study/ |
|  | 61 | Nested case control stud$.tw. |
|  | 62 | **Or/13-61** |
| **Exclusion Criteria** | 63 | exp Case Reports/ |
|  | 64 | “Case report$”.tw. |
|  | 65 | “Case stud$”.tw. |
|  | 66 | “Case series”.tw. |
|  | 67 | exp Comment/ |
|  | 68 | exp Editorial/ |
|  | 69 | Editorial.tw. |
|  | 70 | exp Letter/ |
|  | 71 | Letter.tw. |
|  | 72 | “In vitro”.tw. |
|  | 73 | “Non-alcoholic fatty”.tw. |
|  | 74 | exp Animals/ NOT exp humans/ |
|  | 75 | **Or/63-74** |
| **Total** | **76** | **7 AND 12** |
|  | **77** | **76 AND 62** |
|  | **78** | **77 NOT 75** |
|  | **79** | **REMOVE DUPLICATES FROM 78** |

Supplementary Table 2. Search strategy for The Cochrane Library

| **Term group** | **Search number** | **Search terms** |
| --- | --- | --- |
| **Exposure** | 1 | MeSH descriptor **Alcoholism** explode all trees |
|  | 2 | “Alcohol dependen*” |
|  | 3 | “Alcohol use disorder*” |
|  | 4 | “Alcohol misuse” |
|  | 5 | Alcoholic* |
|  | 6 | Alcoholism |
|  | **7** | **#1 OR #2 OR #3 OR #4 OR #5 OR #6** |
| **Outcome** | 8 | MeSH descriptor **Death** explode all trees |
|  | 9 | Death* |
|  | 10 | MeSH descriptor **Mortality** explode all trees |
|  | 11 | Mortalit* |
|  | **12** | **#8 OR #9 OR #10 OR #11** |
| **Study Design** | 13 | MeSH descriptor **Randomized Controlled Trial** explode all trees |
|  | 14 | MeSH descriptor **Randomized Controlled Trials as Topic** explode all trees |
|  | 15 | MeSH descriptor **Random Allocation** explode all trees |
|  | 16 | MeSH descriptor **Randomization** explode all trees |
|  | 17 | MeSH descriptor **Double Blind Method** explode all trees |
|  | 18 | MeSH descriptor **Single Blind Method** explode all trees |
|  | 19 | ((singl* or doubl* or treb* or tripl*) NEXT (blind* or mask*)) |
|  | 20 | MeSH descriptor **Clinical Trial** explode all trees |
|  | 21 | MeSH descriptor **Clinical Trials as Topic** this term only |
|  | 22 | MeSH descriptor **Clinical Trial, Phase II** explode all trees |
|  | 23 | MeSH descriptor **Clinical Trial, Phase III** explode all trees |
|  | 24 | MeSH descriptor **Clinical Trial, Phase IV** explode all trees |
|  | 25 | MeSH descriptor **Multicenter Study** explode all trees |
|  | 26 | Clinical NEXT trial* |
|  | 27 | Clinical NEXT stud* |
|  | 28 | MeSH descriptor **Placebos** explode all trees |
|  | 29 | Placebo*:ti,ab,kw |
|  | 30 | Randomly NEXT allocated |
|  | 31 | Random NEXT allocation |
|  | 32 | RCT* |
|  | 33 | ((“Randomized controlled”) NEXT (trial* or stud*)) |
|  | 34 | ((“Randomised controlled”) NEXT (trial* or stud*)) |
|  | 35 | ((“Open-label”) NEXT (trial* or stud*)) |
|  | 36 | “Non-blinded stud*” |
|  | 37 | MeSH descriptor **Cohort** **Studies** explode all trees |
|  | 38 | “Cohort analy*” |
|  | 39 | “Cohort stud*” |
|  | 40 | MeSH descriptor **Longitudinal Studies** explode all trees |
|  | 41 | Longitudinal* |
|  | 42 | (Prospective NEXT (cohort* or stud*)) |
|  | 43 | MeSH descriptor **Follow-up** **Studies** explode all trees |
|  | 44 | MeSH descriptor **Prospective Studies** explode all trees |
|  | 45 | MeSH descriptor **Nested Case Control Studies** explode all trees |
|  | 46 | Nested NEXT case* |
|  | 47 | ((“Follow-up”) NEXT (stud*)) |
|  | **48** | **#13 OR #14 OR #15 OR #16 OR #17 OR #18 OR #19 OR #20 OR #21 OR #22 OR #23 OR #24 OR #25 OR #26 OR #27 OR #28 OR #29 OR #30 OR #31 OR #32 OR #33 OR #34 OR #35 OR #36 OR #37 OR #38 OR #39 OR #40 OR #41 OR #42 OR #43 OR #44 OR #45 OR #46 OR #47** |
| **Exclusion Criteria** | 49 | MeSH descriptor **Case Reports** explode all trees |
|  | 50 | “Case report*” |
|  | 51 | “Case stud*” |
|  | 52 | “Case series” |
|  | 53 | MeSH descriptor **Comment** explode all trees |
|  | 54 | MeSH descriptor **Editorial** explode all trees |
|  | 55 | Editorial |
|  | 56 | MeSH descriptor **Letter** explode all trees |
|  | 57 | Letter |
|  | 58 | “In vitro” |
|  | 59 | “Non-alcoholic fatty” |
|  | **60** | **#49 OR #50 OR #51 OR #52 OR #53 OR #54 OR #55 OR #56 OR #57 OR #58 OR #59** |
| **Total** | **61** | **#7 AND #12** |
|  | **62** | **#61 AND #48** |
|  | **63** | **#62 NOT #60** |

Supplementary Table 3. Summary of the critical appraisal of the credibility of studies included in the systematic literature review. Studies deemed ‘not credible’ were excluded from the meta-analyses.

| **Study** | **Design** | **Data** | **Analyses** | **Reporting** | **Interpretation** | **Conflicts of Interest** | **Credible?** |
| --- | --- | --- | --- | --- | --- | --- | --- |
| Bell & Orjasaeter 1983 | **Weakness** - not enough information provided about the study protocol. | **Weakness** - not enough information provided about the definition of AD or the source of the reference population. | **Neutral** - results were reported separately for males and females, but confounding was not considered and sensitivity analyses were not carried out. | **Weakness** - not enough information is given about the calculation of SMRs, and no CIs are reported. | **Neutral** - the results are clear and meaningful but there is little interpretation provided. | **Neutral** - the conflict of interest is unlikely to have affected the results. | **No** |
| Berglund & Tunving 1985 | **Strength** - the study was grant-funded implying a pre-specified plan, and the expected mortality rates were adjusted for age, sex and time period. | **Neutral** - generally very good, but no definition is given for AD. | **Strength** - expected deaths were adjusted for key confounders, but no sensitivity analyses were carried out. | **Neutral** - generally good, but the authors did not provide baseline characteristics or CIs for their SMRs. | **Strength** - the results are discussed in detail. | **Neutral** - the conflict of interest is unlikely to have affected the results. | **Yes** |
| Campos et al. 2011 | **Strength** - the study was grant-funded implying a pre-specified plan, and the expected mortality rates were adjusted for age and sex. | **Strength** - AD is defined and the data sources are described in detail. | **Strength** - hazard ratios were calculated with and without adjustment for key confounders to investigate their effects. | **Strength** - the reporting is clear and thorough. | **Strength** - the results are discussed in detail and put into the context of other studies. | **Neutral** - the conflict of interest is unlikely to have affected the results. | **Yes** |
| Dawson 2000 | **Strength** - the methods are described and justified very thoroughly. | **Strength** - AD is defined and the data sources are described in detail. | **Strength** - hazard ratios were calculated with and without adjustment for key confounders to investigate their effects. | **Strength** - the reporting is clear and thorough. | **Strength** - the results are discussed in detail and put into the context of other studies. | **Neutral** - no conflicts of interest were declared. | **Yes** |
| de Lint & Schmidt 1970 | **Neutral** - the design was adequate but it is unclear whether the study goals or analysis protocol were pre-specified. | **Neutral** - generally very good, but no definition is given for AD. | **Strength** - SMRs were calculated for various subgroups and a sensitivity analysis was carried out to exclude subjects with liver cirrhosis as a primary cause of death. | **Neutral** - generally good, but the authors do not specify what proportion of the sample were male or female and the extent of missing mortality data is unclear. | **Strength** - the discussion is brief but sufficient. | **Neutral** - the conflict of interest is unlikely to have affected the results. | **Yes** |
| de Lint & Levinson 1975 | **Neutral** - the design was adequate but it is unclear whether the analysis protocol was pre-specified. | **Neutral** - generally very good, but the definition of alcohol addiction is unclear. | **Strength** - expected deaths were adjusted for key confounders, but no sensitivity analyses were carried out | **Neutral** - generally good, but the authors did not provide baseline characteristics or confidence intervals for their SMRs | **Strength** - the results are discussed in detail and put into the context of other studies. | **Neutral** - the conflict of interest is unlikely to have affected the results. | **Yes** |
| De Silva & Ellawala 1994 | **Weakness** - it is unclear whether the study goals or analysis protocol were pre-specified. The expected death rates were calculated for men of a single age range, but the subjects included men outside this range. | **Neutral** - generally very good, but the definition of AD is old and subjective. | **Neutral** - the authors briefly consider confounders, but there are no subgroup or sensitivity analyses. | **Neutral** - generally good, but the authors did not report their statistical methods and did not provide confidence intervals for their SMRs. | **Strength** - the discussion is reasonably detailed. | **Weakness** - the study supports the goal of temperance for AD subjects, which is the service provided by the clinic. Without evidence of a pre-specified plan, there is a chance of bias in the protocol. | **Yes** |
| Denison et al. 1997 | **Strength** - the study was grant-funded implying a pre-specified plan, and the expected mortality rates were adjusted for various confounders. | **Strength** - AD is defined and the data sources are described in detail. | **Strength** - expected deaths were adjusted for several confounders, the effects of age were investigated through use of a model, and the authors carried out a sensitivity analysis with revised causes of death. | **Strength** - the reporting is clear and thorough, apart from the lack of descriptive statistics for the cohort as a whole. | **Strength** - the discussion is extensive and detailed, and puts the results into the context of other studies. | **Neutral** - the conflict of interest is unlikely to have affected the results. | **Yes** |
| Feuerlein et al. 1994 | **Neutral** - the design was adequate but it is unclear whether the study goals or analysis protocol were pre-specified. | **Neutral** - generally good, but no definition is given for AD. | **Strength** - the regression models were very detailed and included a wide range of potential confounders. | **Neutral** - generally good, but there is a lack of descriptive statistics and the authors did not provide confidence intervals for their SMRs. | **Strength** - the discussion is extensive and detailed. | **Neutral** - no conflicts of interest were declared. | **Yes** |
| Finney & Moos 1991 | **Strength** - the study was grant-funded implying a pre-specified plan, and controls were matched for various confounding characteristics. | **Neutral** - generally good, but no definition is given for AD. | **Strength** - the authors considered multiple potential confounders. | **Neutral** - some information was missing from the paper, including confidence intervals. | **Strength** - the discussion is detailed and puts the results into the context of other studies. | **Neutral** - no conflicts of interest were declared. | **Yes** |
| Fitzgerald et al. 1971 | **Weakness** - not enough information provided about the study protocol. | **Weakness** - not enough information provided about the definition of AD or the source of the reference population. | **Weakness** - not enough information. | **Weakness** - not enough information is given about the calculation of SMRs, and no confidence intervals are reported. | **Neutral** - the discussion of mortality is limited. | **Neutral** - no conflicts of interest were declared. | **No** |
| Gerdner & Berglund 1997 | **Strength** - the study was grant-funded implying a pre-specified plan, and the expected mortality rates were adjusted for various confounders. | **Neutral** - generally good, but no definition is given for AD. | **Neutral** - brief discussion of confounders, and subgroups by sex and drinking outcome. | **Strength** - the reporting is clear and thorough. | **Strength** - the discussion is detailed and puts the results into the context of other studies. | **Neutral** - the conflict of interest is unlikely to have affected the results. | **Yes** |
| Gillis 1969 | **Neutral** - the design was adequate but it is unclear whether the study goals or analysis protocol were pre-specified, and the general population data are not from the same years as the study. | **Neutral** - generally good, but no definition is given for AD. | **Strength** - potentially confounding characteristics were considered, and SMRs were calculated for age subgroups. | **Neutral** - some information was missing from the paper, including confidence intervals. | **Strength** - the discussion is detailed and puts the results into the context of other studies. | **Neutral** - the conflict of interest is unlikely to have affected the results. | **Yes** |
| Gual et al. 2009 | **Strength** - the study was grant-funded implying a pre-specified plan, and the study used an inception cohort of patients to minimize confounding. | **Strength** - AD is defined and the data sources are described in detail. | **Neutral** - the study would have benefited from a sensitivity analysis concerning patients lost to follow-up to allow greater comparability to other studies. | **Neutral** - as the authors did not calculate relative measures of outcome (eg. RRs), it was not possible to extract confounder-adjusted estimates. | **Strength** - the discussion is detailed and puts the results into the context of other studies. | **Neutral** - no conflicts of interest were declared. | **Yes** |
| Haver et al. 2009 | **Strength** - the study was grant-funded and approved by an ethics committee implying thorough planning, and controls were matched for relevant confounders. | **Neutral** - although all the subjects had alcohol addiction this is not defined in the paper. A good range of data sources was used. | **Strength** - potentially confounding characteristics were considered, and SMRs were calculated for age subgroups. | **Strength** - the reporting is clear and thorough. | **Strength** - the discussion is detailed and puts the results into the context of other studies. | **Neutral** – the conflict of interest is unlikely to have affected the results. | **Yes** |
| Hiroeh et al. 2008 | **Strength** - the study was grant-funded and approved by a regulatory authority, implying a pre-specified plan, and expected mortality was adjusted for relevant confounders. | **Strength** - AD is defined and the data sources (Danish national registries) are known to have very high coverage and linkage. | **Strength** - confounders were considered, SMRs were calculated for sex subgroups, and an important sensitivity analysis was carried out. | **Strength** - the reporting is clear and thorough. | **Strength** - the discussion is detailed and puts the results into the context of other studies. | **Neutral** - the conflict of interest is unlikely to have affected the results. | **Yes** |
| John et al. 2013 | **Strength** - this is part of a larger study which involved a large general population survey. Expected mortality was adjusted for relevant confounders. | **Strength** - AD is defined and the data sources are described in detail. | **Strength** - mortality rates were presented for a number of subgroups, and an important sensitivity analysis was carried out. | **Strength** - the reporting is clear and very thorough. | **Strength** - the discussion is detailed and puts the results into the context of other studies. | **Neutral** - no conflicts of interest were declared. | **Yes** |
| Johnson 2001 | **Strength** - the study was grant-funded and ethically approved, implying a pre-specified plan, and expected mortality was adjusted for relevant confounders. | **Strength** - AD is defined and the data sources are sufficient. | **Neutral** - confounders were discussed, but no subgroup analyses or sensitivity analyses were carried out. | **Strength** - the reporting is thorough. | **Strength** - the discussion is brief but sufficient, and highlights several of the limitations of the study. | **Neutral** - no conflicts of interest were declared. | **Yes** |
| Kessel & Grossman 1961 | **Neutral** - the design was adequate but it is unclear whether the hypothesis or analysis plan were pre-specified. | **Neutral** - AD is not defined, and data sources were insufficient for the St Pancras series. | **Neutral** - limited analysis of confounders. | **Neutral** - somewhat limited information on the Maudsley series, and very limited information on the St Pancras series. | **Neutral** - the discussion is brief and does not consider confounders. | **Neutral** - no conflicts of interest were declared. | **No** |
| Mackenzie et al. 1986 | **Neutral** - the design was generally good, but the expected mortality was based on general population data from just three years out of the eight years of follow-up. | **Neutral** - no definition of AD is given. | **Strength** - a regression model was used to assess a variety of potential confounders. | **Strength** - although confidence intervals were not given, the reporting was generally clear and thorough. | **Strength** - the discussion is detailed and puts the results into the context of other studies. | **Neutral** - the conflict of interest is unlikely to have affected the results. | **Yes** |
| Markkula et al. 2012 | **Strength** - the study was grant-funded, implying a pre-specified plan, and the use of a general population survey minimized bias in the results. | **Strength** - AD is defined, and the data sources are reliable. | **Strength** - four regression models were built to examine potential confounders in various combinations, although this was only done for the group-level analysis (alcohol use disorders) and not for AD specifically. | **Strength** - the reporting was clear and thorough, although relative measures were not reported for the dependence vs abuse comparison. | **Strength** - the discussion is detailed and puts the results into the context of other studies. | **Neutral** - the conflict of interest is unlikely to have affected the results. | **Yes** |
| Marshall et al. 1994 | **Strength** - the study was grant-funded, implying a pre-specified plan, and expected mortality was adjusted for relevant confounders. | **Neutral** - no definition of AD is given, although the subjects are split into 'moderately dependent' and 'severely dependent' | **Strength** - there was some assessment of confounders, and subgroups by level of dependence (moderate vs severe). | **Strength** - although confidence intervals were not given, the reporting was generally clear and thorough. | **Strength** - the discussion is sufficient and puts the results into the context of other studies. | **Neutral** - the conflict of interest is unlikely to have affected the results. | **Yes** |
| Martin et al. 1985a | **Strength** - the study was grant-funded, implying a pre-specified plan, and expected mortality rates were adjusted for the key potential confounders. | **Strength** - AD is defined and the data sources are reliable. | **Neutral** - there was a thorough assessment of confounders, although it was not carried out for AD patients specifically. | **Strength** - although confidence intervals were not given, the reporting was generally clear and thorough. | **Strength** - the discussion is detailed and puts the results into the context of other studies. | **Neutral** - the conflict of interest is unlikely to have affected the results. | **Yes** |
| Martin et al. 1985b | **Strength** - the study was grant-funded, implying a pre-specified plan, and expected mortality rates were adjusted for the key potential confounders. | **Strength** - AD is defined and the data sources are reliable. | **Neutral** - there was a thorough assessment of confounders, although it was not carried out for alcohol-dependent patients specifically. | **Strength** - although confidence intervals were not given, the reporting was generally clear and thorough. | **Strength** - the discussion is detailed and puts the results into the context of other studies. | **Neutral** - the conflict of interest is unlikely to have affected the results. | **Yes** |
| Mattisson et al. 2011 | **Strength** - the study was based on a large general population survey, and involved an inception cohort of alcohol-dependent subjects to minimize confounding. | **Strength** - AD is defined, although some diagnoses were applied retrospectively, and the data sources are extensive and reliable. | **Strength** - simple and multivariate Cox regression models were used to investigate a number of potential confounders. | **Strength** - the reporting was clear and thorough, although the absolute number of deaths was not reported separately for the dependence and abuse groups. | **Strength** - the discussion is detailed, explicitly considers the strengths and limitations of the study, and puts the results into the context of previous studies. | **Strength** - no conflicts of interest were identified. | **Yes** |
| Min et al. 2008 | **Strength** - the authors state that this analysis was not planned at the time of the baseline study, but that the analysis plan was developed before carrying out the follow-up survey. Statistical power was explicitly considered in the design of the study. | **Strength** - AD is defined, but it is unclear how deaths were identified or confirmed. | **Strength** - six different Cox regression models were built to examine the effects of a number of confounders. | **Strength** - the reporting is clear and very thorough, with multiple adjusted ORs presented. | **Strength** - the discussion is detailed, explicitly considers the strengths and limitations of the study, and puts the results into the context of previous studies. | **Neutral** - the conflict of interest is unlikely to have affected the results. | **Yes** |
| Moos et al. 1994 | **Strength** - the study was grant-funded, implying a pre-specified plan, and expected mortality rates were adjusted for the key potential confounders. | **Strength** - AD is defined and the data sources are extensive. | **Neutral** - the analysis is extensive, but alcohol-dependent subjects were a subgroup of the results so there were no subgroup or sensitivity analyses for the alcohol-dependent group specifically. | **Strength** - the reporting is clear and thorough, although 95% CIs were not reported for SMRs. | **Strength** - the discussion is detailed, explicitly considers the strengths and limitations of the study, and puts the results into the context of previous studies. | **Neutral** - the conflict of interest is unlikely to have affected the results. | **Yes** |
| Murphy et al. 2008 | **Strength** - the study was grant-funded, implying a pre-specified plan, and expected mortality rates were adjusted for the key potential confounders. | **Neutral** - AD is not defined, but the data sources are generally very good. | **Strength** - Cox regression models were used to examine the effects of various confounders separately and individually. | **Neutral** - the number of subjects reassessed at the 16-year follow-up was not specified and the absolute number of patients with AD was not given. | **Strength** - the discussion puts the results into the context of previous studies and discusses the changes over time since the baseline survey in 1952, although unmeasured confounding is not discussed. | **Neutral** - no conflicts of interest were declared. | **Yes** |
| Neumark et al. 2000 | **Strength** - the study was grant-funded, implying a pre-specified plan, and expected mortality rates were adjusted for the key potential confounders. | **Strength** - AD is defined, and the data sources are adequate. | **Strength** - Cox regression models were used to examine the effects of various confounders, and sensitivity analyses were carried out to assess the effects of excluding subjects lost to follow-up and grouping subjects by 1-year age group instead of 10-year groups. | **Strength** - the reporting is clear and thorough, although the absolute number of deaths for the alcohol-dependent group and the alcohol abuse group were not reported. | **Strength** - the discussion is detailed, explicitly considers the strengths and limitations of the study, and puts the results into the context of previous studies. | **Neutral** - no conflicts of interest were declared. | **Yes** |
| Noda et al. 2001 | **Strength** - the study was grant-funded and received prior approval, implying a pre-specified plan, and expected mortality rates were adjusted for potential confounders. | **Strength** - AD is defined, although the definition is in Japanese so it is not possible to assess its similarities to DSM or ICD criteria, and multiple data sources were used. | **Strength** - potentially confounding characteristics were considered, SMRs were calculated for subgroups by treatment type, and a sensitivity analysis was performed to examine the effects of excluding patients who were followed up for less than a year. | **Strength** - the reporting is clear and mostly thorough, although subgroups by length of follow-up are not reported for all causes of death and this decision is not justified in the paper. | **Strength** - the discussion is detailed and thorough, although there appears to be some cherry-picking of subgroup analyses to draw conclusions from. | **Neutral** - no conflicts of interest were declared. | **Yes** |
| Pell & D’Alonzo 1973 | **Neutral** - this is one of a series of studies performed to measure the long-term effects of excessive drinking on health and longevity among employees of a single company. Alcohol-dependent subjects were identified during their medical check-ups with the company, but this method is likely to have missed a number of subjects who might want to conceal their addiction from their employer. | **Weakness** - the data sources may not have been sufficient as no independent sources were included, and the definition of AD was based on subjective observations. | **Strength** - various potential confounders were considered and explored through subgroup analyses. | **Strength** - the reporting was thorough, although unfortunately the scanned copy of the article was of low quality so some data were illegible. | **Strength** - the discussion is thorough. | **Weakness** - the subjects of this study were employees of a single company, and analysis was carried out by members of the company's medical division (presumably with company funding), which may have biased the results in either direction. | **Yes** |
| Perala et al. 2010 | **Strength** - the study was grant-funded and received prior ethical approval implying a pre-specified plan, and the sample was drawn from a representative general population survey to minimize confounding. | **Strength** - the data sources were extensive and reliable, and AD is defined. | **Strength** - various potential confounders were considered and explored through subgroup analyses, although these were only performed for subjects with alcohol-induced psychotic syndrome (AIPS) and not AD more widely. Sensitivity analyses were performed to ensure that no data points were outliers. | **Strength** - the reporting was clear and thorough. | **Strength** - the discussion is thorough and puts the results in the context of other studies. | **Strength** - the authors state that there were no conflicts of interest. | **Yes** |
| Poser et al. 1992 | **Neutral** - the study was grant-funded, implying a pre-specified plan, but it is not clear from the paper whether the expected mortality rates were adjusted for age and sex. | **Strength** - the data sources were extensive and reliable, and AD is defined. | **Neutral** - although confounders were considered and subgroup analyses were carried out, these were not done for the alcohol-dependent group specifically. | **Neutral** - the reporting was generally good, but it was not clear whether the SMRs were confounder-adjusted, and the absolute number of deaths was not reported for the alcohol-dependent subjects specifically. | **Strength** - the discussion was thorough, although the discussion of AD specifically was limited. | **Neutral** - no conflicts of interest were declared. | **Yes** |
| Rankin et al. 1970 | **Neutral** - there is not enough information to judge whether the protocol was pre-specified. | **Weakness** - AD is not defined, and there is no information on how causes of death were assessed. | **Strength** - subgroup analyses were used to examine the effects of various potential confounders. | **Neutral** - the authors did not carry out any statistical analysis on the mortality data with respect to drinking outcome, so it was not possible for them to present confounder-adjusted estimates or confidence intervals. However, the raw data were presented clearly. | **Strength** - the discussion is thorough and puts the results in the context of other studies. | **Neutral** - the conflict of interest is unlikely to have affected the results. | **Yes** |
| Saieva et al. 2012 | **Strength** - although it is not clear whether the analysis plan was pre-specified, the design was generally very good. | **Strength** - the data sources were comprehensive and reliable, and AD is defined. | **Strength** - various potential confounders were considered, and SMRs were calculated separately for males and females in a subgroup analysis. | **Strength** - the reporting was clear and thorough. | **Strength** - the discussion is very thorough, and considers a number of potential confounders relating to the study participants and the general population of the surrounding area. | **Neutral** - no conflicts of interest were declared. | **Yes** |
| Schmidt & de Lint 1969 | **Strength** - although it is not clear whether the analysis plan was pre-specified, the design was generally very good. | **Neutral** - the data sources were good, but there is no definition of AD given in the paper. | **Strength** - the confounding effects of age and sex were examined through subgroup analyses and a sensitivity analysis. | **Neutral** - patient flow (including the number of individuals screened for selection in the cohort and the extent of missing data) was unclear and confidence intervals were not provided for SMRs, but there is evidence that they were calculated by the authors. | **Strength** - the discussion is brief but covers the key points. | **Neutral** - the conflict of interest is unlikely to have affected the results. | **Yes** |
| Smith et al. 1983 | **Strength** - the study was grant-funded, implying a pre-specified analysis plan, and interviews about alcohol use were corroborated by also interviewing all first-degree relatives of each subject. | **Strength** - the data sources were good, and AD was defined. | **Strength** - the effects of various confounders were assessed through subgroup analyses and discriminant analyses. | **Strength** - the reporting was generally good, although 95% CIs were not reported. | **Weakness** - confidence intervals were not calculated, and this led to an over-interpretation of key results regarding drinking status and mortality. | **Weakness** - the authors are reporting on patients from their own facilities, which promote abstinence, and the results were over-interpreted in support of abstinence as a treatment goal. | **Yes** |
| Storbjörk & Ullman 2012 | **Strength** - the design was carefully considered to minimize confounding, and the study's prior ethical approval implies a pre-specified analysis plan. | **Strength** - the data sources were good, and AD was defined. | **Strength** - three regression models were built to test the effects of various confounders, and the interactions between alcohol and drug dependence were tested. | **Strength** - the reporting was exceptionally thorough. | **Strength** - the authors relate the results to their four original hypotheses, and discuss the findings in the context of previous studies. | **Strength** - the authors state that there were no conflicts of interest. | **Yes** |
| Tashiro & Lipscomb 1963 | **Strength** - although it is not clear whether the analysis plan was pre-specified, the design was generally very good. | **Neutral** - the data sources were good, but there is no definition of AD given in the paper. | **Neutral** - the authors state that there were no significant differences from the general population mortality rates for various subgroups, but the expected mortality rates are not reported in the paper. | **Neutral** - the authors report only approximate SMRs (eg. "nearly 2.5 times greater than the expected average annual death rate") without CIs, and it is unclear how many patients declined to participate at baseline. | **Strength** - the discussion is brief but covers the key points. | **Neutral** - no conflicts of interest were declared. | **Yes** |
| Thorarinsson 1979 | **Strength** - the study was grant-funded, implying a pre-specified plan, SMRs were adjusted for key confounders, and certified causes of death were used in all cases to ensure comparability with general population data. | **Neutral** - the data sources were good, but there is no definition of AD given in the paper. | **Strength** - the confounding effects of age were assessed, and the differences in mortality between outpatients, inpatients and outpatient-inpatients were examined through subgroup analyses. | **Strength** - the reporting was generally good, although 95% CIs were not reported. | **Strength** - the authors discuss the results in the context of a number of confounders, although they do not thoroughly assess the possible effects of each one. | **Neutral** - no conflicts of interest were declared. | **Yes** |
| Vaillant et al. 1983 | **Strength** - the study was grant-funded, implying a pre-specified plan, and the authors specifically designed a long-term prospective study to minimize the confounding effects seen in very short-term studies. | **Strength** - numerous data sources were used, and the operational definitions of alcohol withdrawal, stable remission, intermittent alcohol abuse and chronic AD were reported in the paper. | **Strength** - the effects of a number of confounders were examined. | **Neutral** - the authors did not present confounder-adjusted estimates or confidence intervals, but they did carry out chi-squared tests to measure significance. | **Strength** - the authors discuss the results in the context of previous studies, and provide recommendations for treatment programmes based on their findings. | **Neutral** - the conflict of interest is unlikely to have affected the results. | **Yes** |
| Vaillant 2003 | **Strength** - this analysis was part of a long-term study on two separate cohorts, and the authors carried out analyses on the two cohorts separately to minimize confounding. | **Strength** - each cohort was followed up regularly with questionnaires and physical examinations, so the data sources were comprehensive, and AD was defined. | **Neutral** - subgroup analyses were carried out by drinking outcome, but potential confounders for mortality specifically were not considered. | **Neutral** - the authors did not carry out any statistical analysis on the mortality data with respect to drinking outcome, so it was not possible for them to present confounder-adjusted estimates or confidence intervals. However, the raw data were presented clearly. | **Strength** - the discussion considers the limitations of the study and puts the results into the context of previous studies. | **Neutral** - no conflicts of interest were declared. | **Yes** |
| Wallerstedt et al. 1995 | **Strength** - the study was grant-funded, implying a pre-specified plan, and the study used an inception cohort followed up prospectively to minimize confounding. | **Strength** – although DSM-III criteria were not used for defining AD subjects, the authors state that definition used is similar to DSM-III AD. | **Neutral** - confounders were discussed, but no subgroup analyses or sensitivity analyses were carried out due to the small sample size. | **Neutral** - statistical analyses were carried out, but it was unclear whether these were hazard ratios or risk ratios. Baseline characteristics were not reported. | **Strength** - the results are discussed in the context of other studies. | **Neutral** - the conflict of interest is unlikely to have affected the results. | **Yes** |
| Wells & Walker 1990 | **Strength** - the study was grant-funded, implying a pre-specified plan, and expected mortality rates were adjusted for age and sex. | **Neutral** - the data sources were good, but there is no definition of AD given in the paper. | **Strength** - the effects of age and sex were analysed and discussed in detail. | **Strength** - the reporting was clear and thorough. | **Strength** - the discussion is detailed, and where the results differ from other studies the differences are thoroughly discussed with reference to the subgroup analyses. | **Neutral** - there was potential for the results to be biased because the authors are reporting on patients from their own treatment centre and they find a much lower SMR than was found in other studies, but they explain a number of possible reasons for this relating to the patients' baseline characteristics rather than implying that it is due to their own work. | **Yes** |
| Yoshino et al. 1997 | **Neutral** - it is unclear whether this follow-up of an earlier study was pre-planned, and for the analysis of interest the design was not structured to minimize confounding. | **Neutral** - AD was defined, but deaths were reported from informants and not verified using official records. | **Neutral** - some confounders were assessed, but this was not carried out for alcohol-dependent subjects specifically. | **Neutral** - due to the non-hierarchical nature of AD and alcohol abuse definitions in the DSM-III, some subjects in the study were defined as having both abuse and dependence, meaning that the analyses carried out in the paper were not relevant to this study; however, from the data presented it was possible to calculate relevant measures of association. | **Strength** - the discussion puts the results into the context of previous studies. | **Neutral** - the conflict of interest is unlikely to have affected the results. | **Yes** |

AD, alcohol dependence; AIPS, alcohol-induced psychotic syndrome; CI, confidence interval; DSM, Diagnostic and Statistical Manual of Mental Disorders; ICD, International Classification of Diseases; OR, odds ratio; RR, relative risk; SMR, standardised mortality ratio;

**Supplementary Figure 1. Subgroup analysis of studies reporting on all-cause mortality for alcohol-dependent subjects vs the general population**


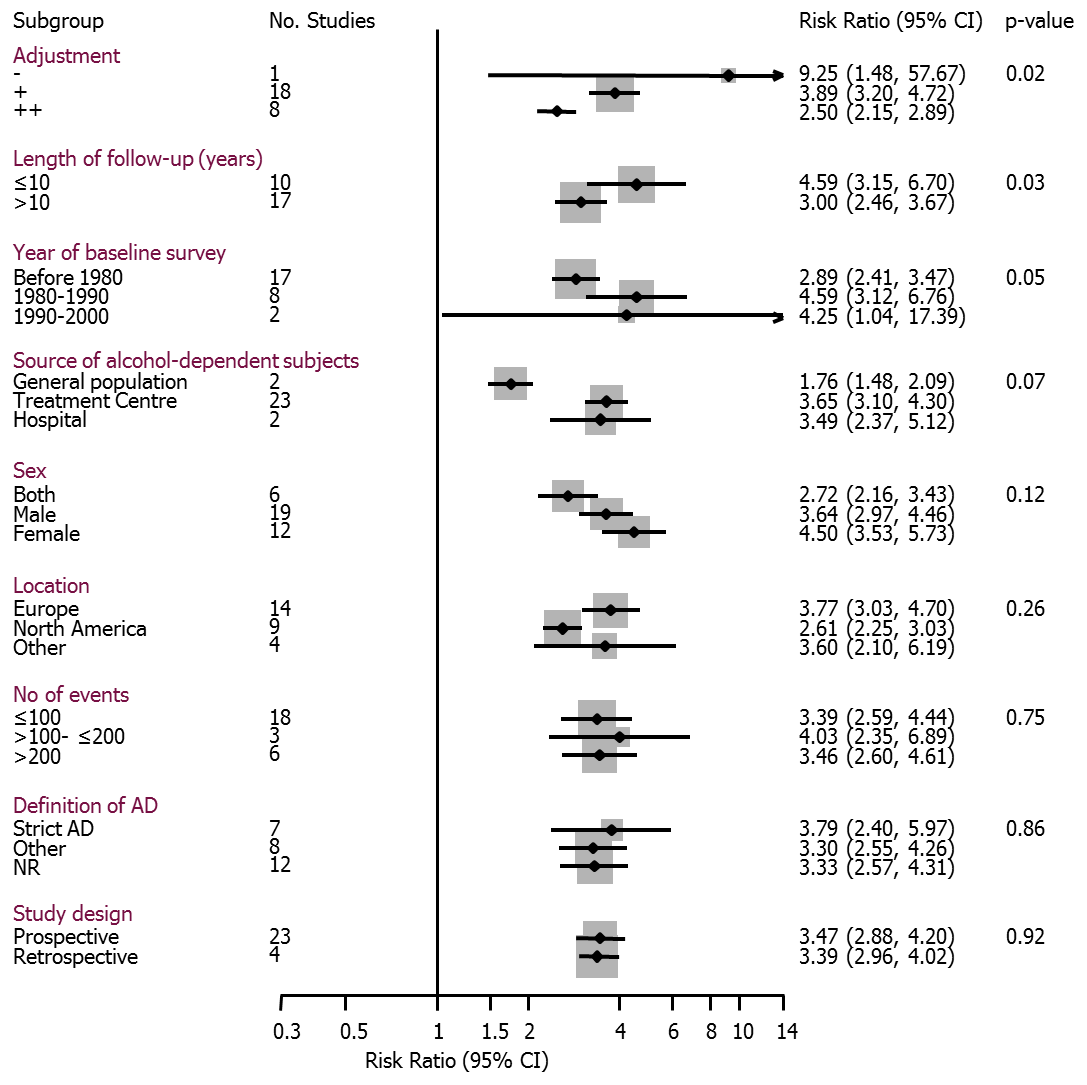


**Supplementary Figure 2. Funnel plots to assess publication bias in studies included in the meta-analysis**


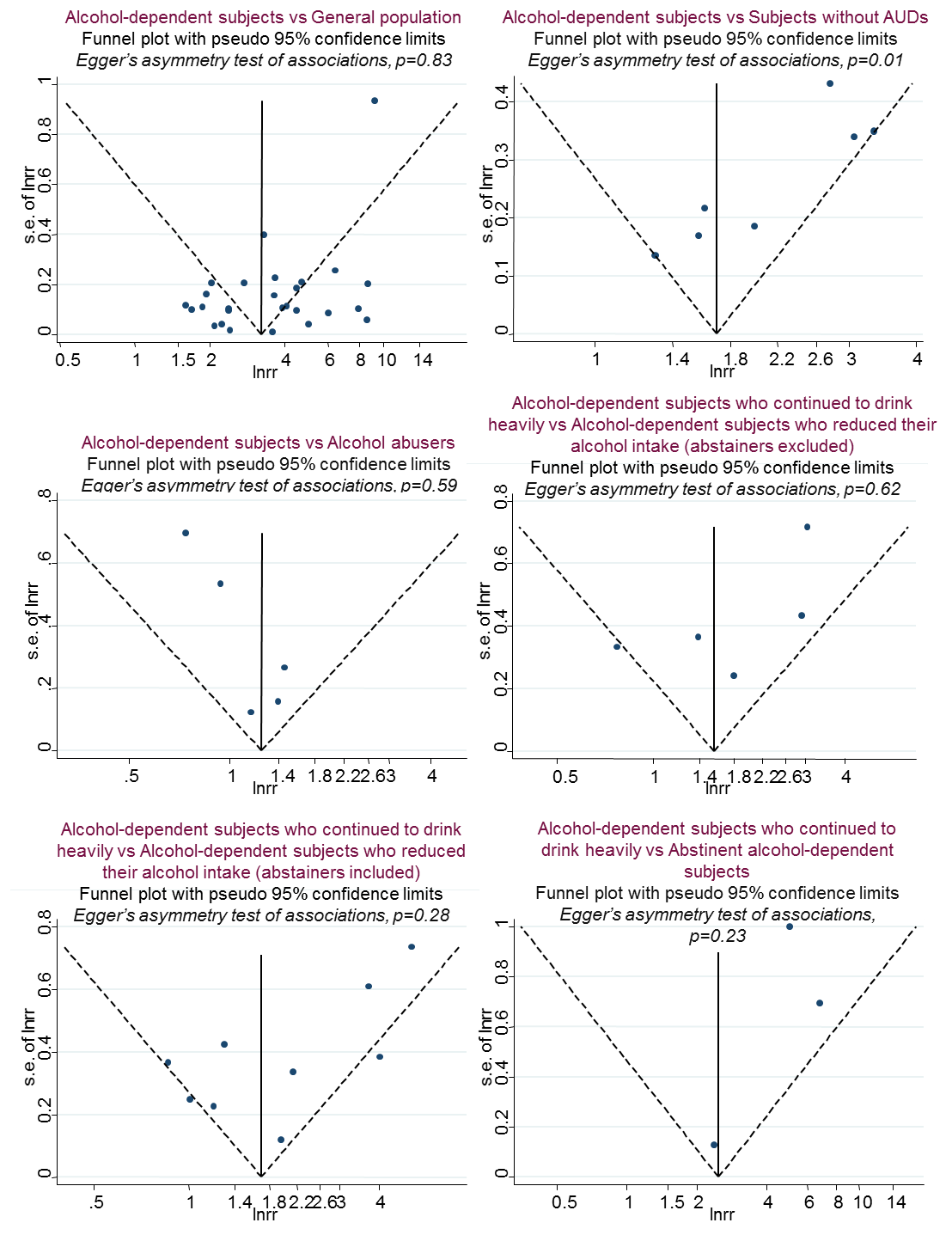

Supplement: Supplementary file 1 — Supplementary material. [file mmc1.docx]
